# Dietary glucose regulates yeast consumption in adult *Drosophila* males

**DOI:** 10.3389/fphys.2014.00504

**Published:** 2014-12-22

**Authors:** Sébastien Lebreton, Peter Witzgall, Marie Olsson, Paul G. Becher

**Affiliations:** ^1^Unit of Chemical Ecology, Department of Plant Protection Biology, Swedish University of Agricultural SciencesAlnarp, Sweden; ^2^Unit of Plant Product Quality, Department of Plant Breeding, Swedish University of Agricultural SciencesAlnarp, Sweden

**Keywords:** adipokinetic hormone, feeding behavior, insulin, starvation, sugar

## Abstract

The adjustment of feeding behavior in response to hunger and satiety contributes to homeostatic regulation in animals. The fruit fly *Drosophila melanogaster* feeds on yeasts growing on overripe fruit, providing nutrients required for adult survival, reproduction and larval growth. Here, we present data on how the nutritional value of food affects subsequent yeast consumption in *Drosophila* adult males. After a period of starvation, flies showed intensive yeast consumption. In comparison, flies stopped feeding after having access to a nutritive cornmeal diet. Interestingly, dietary glucose was equally efficient as the complex cornmeal diet. In contrast, flies fed with sucralose, a non-metabolizable sweetener, behaved as if they were starved. The adipokinetic hormone and insulin-like peptides regulate metabolic processes in insects. We did not find any effect of the adipokinetic hormone pathway on this modulation. Instead, the insulin pathway was involved in these changes. Flies lacking the insulin receptor (InR) did not respond to nutrient deprivation by increasing yeast consumption. Together these results show the importance of insulin in the regulation of yeast consumption in response to starvation in adult *D. melanogaster* males.

## Introduction

The mechanisms controlling food intake and energy homeostasis are fairly well-conserved across animal species with a good homology between vertebrates and invertebrates. After a nutrient-rich meal, calorie intake is detected and induces a feedback that triggers satiety and suppresses food intake through hormonal pathways. In insects, the insulin-like peptide/adipokinetic hormone (ILP/AKH) pathway, homologous to the mammalian insulin/glucagon pathway, plays a central role in feeding regulation (Lee and Park, 2004; Bharucha et al., 2008; Root et al., 2011).

In *Drosophila melanogaster*, food intake promotes the release of Unpaired 2 (Upd2) from the fat body, a protein thought to be homologous to mammalian leptin (Rajan and Perrimon, 2012). Upd2 stimulates the release of ILPs (Rajan and Perrimon, 2012) that act on olfactory neurons by inhibiting the short neuropeptide F (sNPF, a homolog of the neuropeptide Y) pathway, resulting in a decrease in sensitivity to food-related odors (Root et al., 2011). In contrast to feeding, starvation increases physiological sensitivity and induces food intake in insects (Farhadian et al., 2012; Farhan et al., 2013). Increased feeding is at least partly due to the activity of dopaminergic neurons in the sub-esophageal ganglion, the primary taste center (Inagaki et al., 2012; Marella et al., 2012). The release of dopamine acts on taste neurons responsible for sugar sensitivity, which also express AKH receptors (Bharucha et al., 2008).

Food deprivation and dietary macronutrient composition affect both physiology and behavior in *D. melanogaster* larvae (Anagnostou et al., 2010; Andersen et al., 2010; Bjordal et al., 2014) and adults (Skorupa et al., 2008; Becher et al., 2010; Lushchak et al., 2011; Lebreton et al., 2012). Dietary proteins activate both ILP and AKH pathways, whereas dietary sugars activate the ILP pathway but inhibit the AKH pathway (Buch et al., 2008). ILPs and AKH are not only involved in the regulation of feeding, but also in several hunger/satiety-dependent behavioral and physiological traits in adults such as energy mobilization and storage, nutrient absorption in the midgut, fecundity, and reproduction or sexual behavior (Lee and Park, 2004; Bharucha et al., 2008; Liu et al., 2009; Wigby et al., 2011; Kodrík et al., 2012). The effect of a protein-rich diet on these hormonal pathways is well-described (Buch et al., 2008). Yeasts play an essential role in the fly's nutrition and ecology (Begon, 1982; Becher et al., 2012) for which they are an essential source of proteins (Skorupa et al., 2008) and also lipids (Bos et al., 1976). Consequently, one can expect flies fed on a diet lacking these nutrients to be attracted to yeasts even if their previous diet has been of high caloric value.

Despite the ecological importance of yeast, little is known about the physiological factors regulating yeast-feeding behavior in adult *D. melanogaster*. In this study, we investigated whether the lack of essential nutrients in a diet would affect subsequent yeast feeding behavior in *D. melanogaster* males. Dietary glucose was sufficient to modulate appetite and we therefore investigated the physiological and hormonal mechanisms underlying the effect of dietary sugar on further yeast consumption with a focus on the ILP/AKH pathway.

## Materials and methods

### Fly strains and rearing

**Figure 1:**
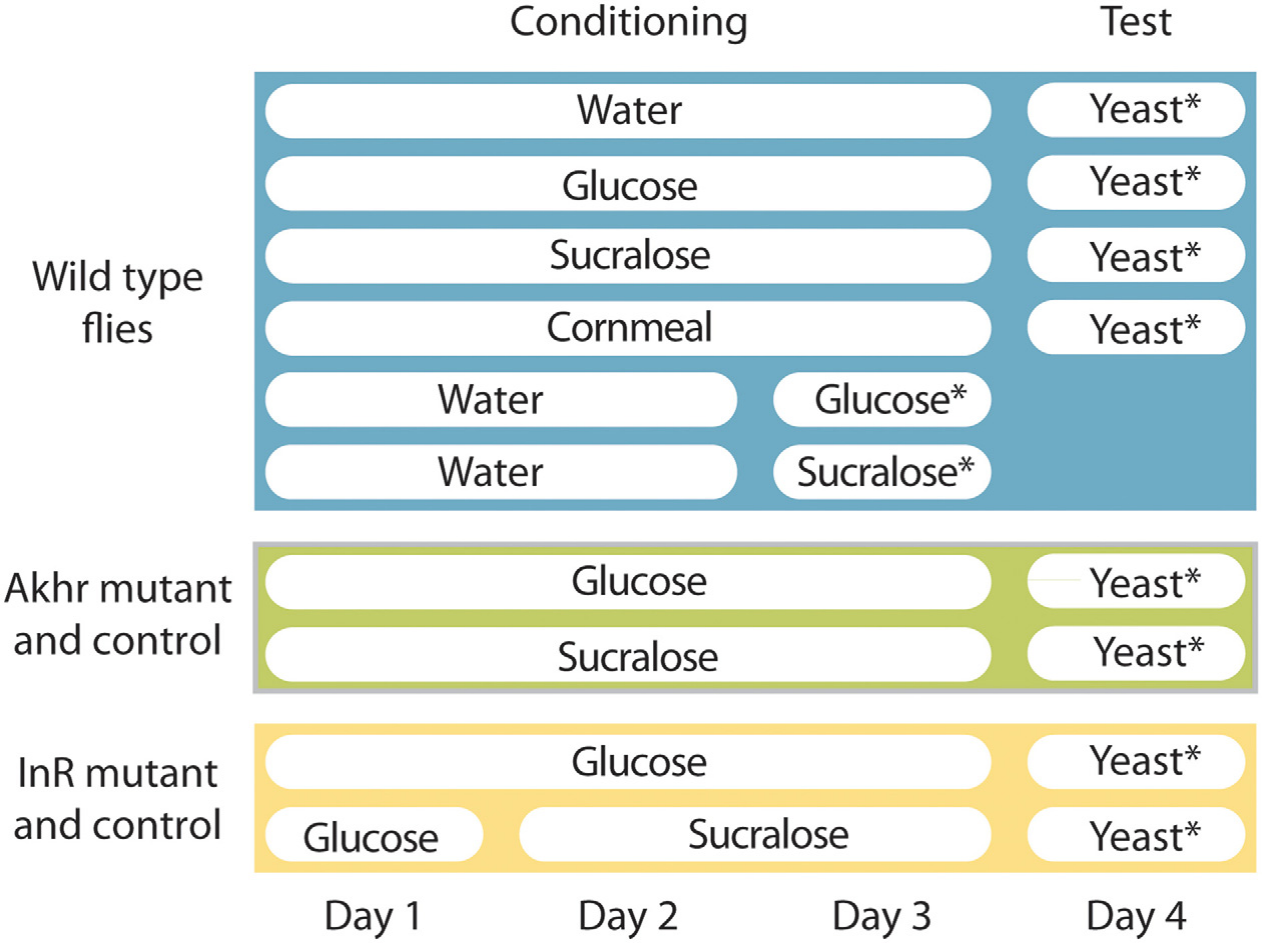
**Overview of the experimental design**. Asterisks indicate when food consumption was measured.

The Dalby strain (Ruebenbauer et al., 2008) of the fruit fly *D. melanogaster* was used as a wild-type (WT) strain. To study the effect of AKH receptor on feeding behavior, an AKHR mutant strain (*Akhr*^Null^) was used and compared to a control (*Akhr*^Rev^) strain (Bharucha et al., 2008). To study the effect of the insulin receptor (InR), two transgenic lines were used: InR^[E19]^/TM2 and InR^GC25^/TM3 (Shingleton et al., 2005). These two lines were crossed and the resulting trans-heterozygous InR^[E19]^/InR^GC25^ was a temperature sensitive mutant of InR (Shingleton et al., 2005). InR^GC25^/TM2 flies were used as a control. The mutant shows a mutant phenotype only when the temperature is raised to 24–25°C (Shingleton et al., 2005). To avoid an effect of the lack of InR during larval development which leads to severe developmental defects, InR^[E19]^/InR^GC25^ and InR^GC25^/TM2 flies were kept at 17°C until adult emergence and then placed at 24°C after emergence during the 3 days preceding behavioral tests. The other flies were reared at room temperature (19–21°C). All flies were reared on a standard sugar-yeast-cornmeal diet [containing corn meal (6%), sugar syrup (6%), malt (1.7%), yeast (1.4%), and soy meal (0.8%)] and under a 12:12 h L:D photoperiod. Newly emerged flies were anesthetized using CO_2_ and sexed under a microscope. Flies of both sexes were then kept in 30 ml plastic tubes with fresh standard cornmeal diet, with only a humidified piece of cotton wool (starved) or a humidified piece of cotton wool supplemented with a 0.5 mL Eppendorf tube filled with a 100 mM solution of a metabolizable (glucose) or non-metabolizable (sucralose) sugar (Figure 1). In order to combat a higher mortality under starvation at 24°C, sucralose-fed InR^[E19]^/InR^GC25^ and InR^GC25^/TM2 flies were kept 1 day with glucose before they were placed with sucralose for the 2 subsequent days (Figure 1). Flies were kept for 3 days before behavioral tests.

### Yeast culture

*Saccharomyces cerevisiae* (S288c) was grown on a synthetic minimal medium (Merico et al., 2007), in 100-mL flasks at 25°C on a rotative shaker for 21 h. The cell suspension was divided into portions and kept at −80°C until application in the feeding assay.

### Feeding assay for yeast consumption

Yeast consumption was analyzed with a modified version of the CAFE assay (Ja et al., 2007; Farhadian et al., 2012). Individual males were isolated and placed in a 30-mL plastic cup. The plastic cup was placed on the top of a 250-mL glass bottle filled with 20 mL of distilled water. The bottom of the cup was pierced to provide humidity. The yeast solution was provided from the top in a 5-μL glass micro-capillary held by a cut pipette tip. Yeast consumption was measured every hour for the first 7 h and after 24 h (Figure 1). A control experiment without flies was conducted at the same time to measure the evaporation rate of the yeast solution, which was used to correct the consumption rate in the experiments with flies.

In order to check the amount of sugar (glucose, sucralose) consumed by flies, the same assay was used with 2-d-old starved mated males over 24 h. Nine to eleven flies were used for each test (Figure 1).

### Statistical analysis

In order to test the differences between different diets (water, cornmeal, glucose, sucralose) on yeast feeding over 24 h, a multiple comparison test (glht function, package multcomp) was performed on the Mixed-Effects Model with individuals and time as random effects (lme function, package nlme). The factor effects were analyzed with an *F*-test. Effect of AKHR and InR on yeast consumption were also analyzed using a Mixed-Effects Model. Mortality of InR flies was tested using a GLM with a binomial distribution (effect of diet and genotype). A χ^2^-test was used to compare mutant and control flies for each diet. Statistical analyses were calculated using R (R 2.1.1, R Development Core Team, Free Software Foundation Boston, MA, USA).

## Results

### Dietary glucose modulates yeast-feeding behavior

**Figure 2:**
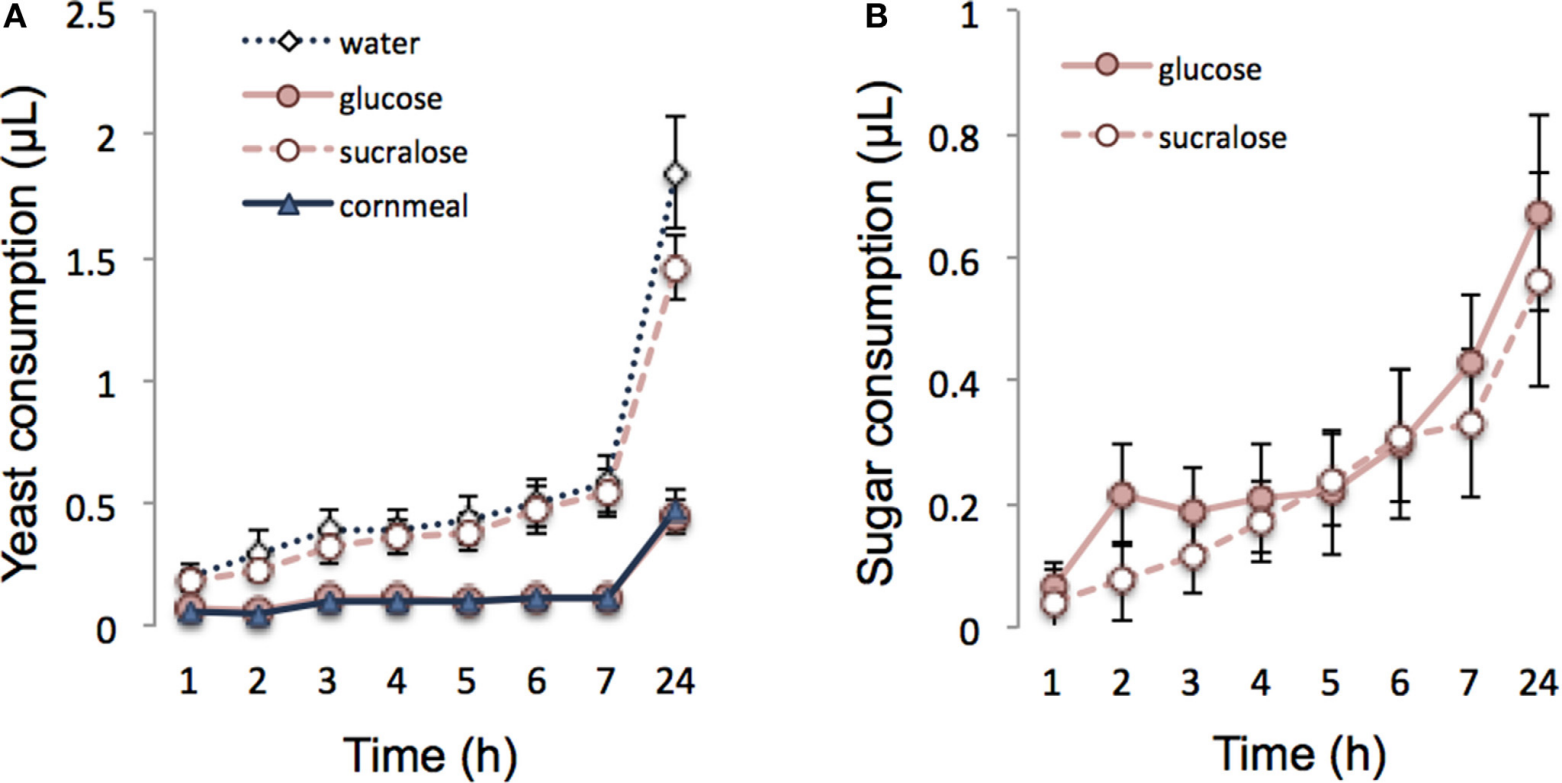
**Modulation of yeast consumption. (A)** Effect of glucose, sucralose, water, and cornmeal diet on subsequent yeast feeding in *Drosophila* during 24 h. **(B)** Consumption of glucose and sucralose by 2-d-old starved flies. All data are mean ± SEM.

The effect of diet on yeast consumption was measured in flies that had access to cornmeal, glucose, sucralose, or water prior to the test. Yeast consumption was significantly affected by the diet (*F* = 10.98, *df* = 3, *p* < 0.0001). There was no difference between sucralose fed and starved flies (Figure 2A; *z* = −0.841, *p* = 0.83). Sucralose-fed and starved flies ate a larger amount of yeast than glucose-fed (sucralose: *z* = 3.93, *p* = 0.003; starved: *z* = 4.45, *p* < 0.001) and cornmeal-fed males (sucralose: *z* = −3.539, *p* = 0.003; starved: *z* = −4.5, *p* < 0.001). On the other hand, glucose fed and cornmeal medium fed flies showed a similar feeding behavior (*z* = −0.047, *p* = 0.99). The difference between glucose- and sucralose-fed flies was not due to a difference in the quantity of sugar consumed since flies ate as much sucralose as glucose solution prior to yeast feeding (Figure 2B; *F* = 17, *df* = 1, *p* = 0.63).

### The insulin pathway influences yeast consumption

**Figure 3:**
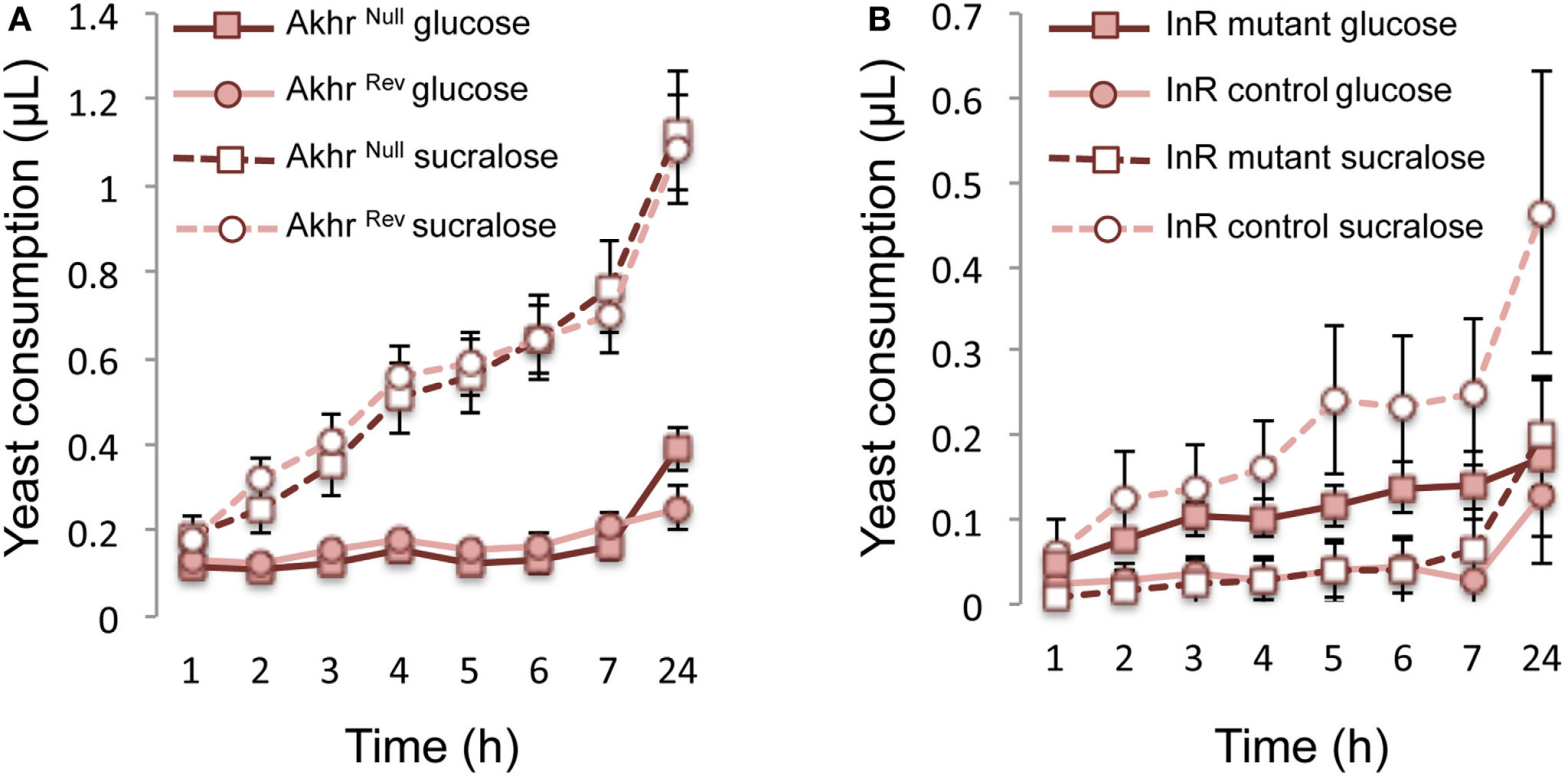
**Hormonal regulation of yeast feeding behavior. (A)** Role of AKH and **(B)** insulin pathway on yeast consumption, in response to dietary sugars. All data are mean ± SEM.

We then studied whether the Insulin/AKH pathway is involved in the effect of glucose on further feeding behavior. The lack of the AKH receptor in *Akhr*^Null^ flies had no effect on yeast consumption when compared to control flies (*Akhr*^Rev^) (*F* = 0.029, *df* = 1, *p* = 0.87), regardless of the diet (glucose or sucralose) (Figure 3A; interaction diet^*^genotype: *F* = 0.00006, *df* = 1, *p* = 0.99).

**Figure 4:**
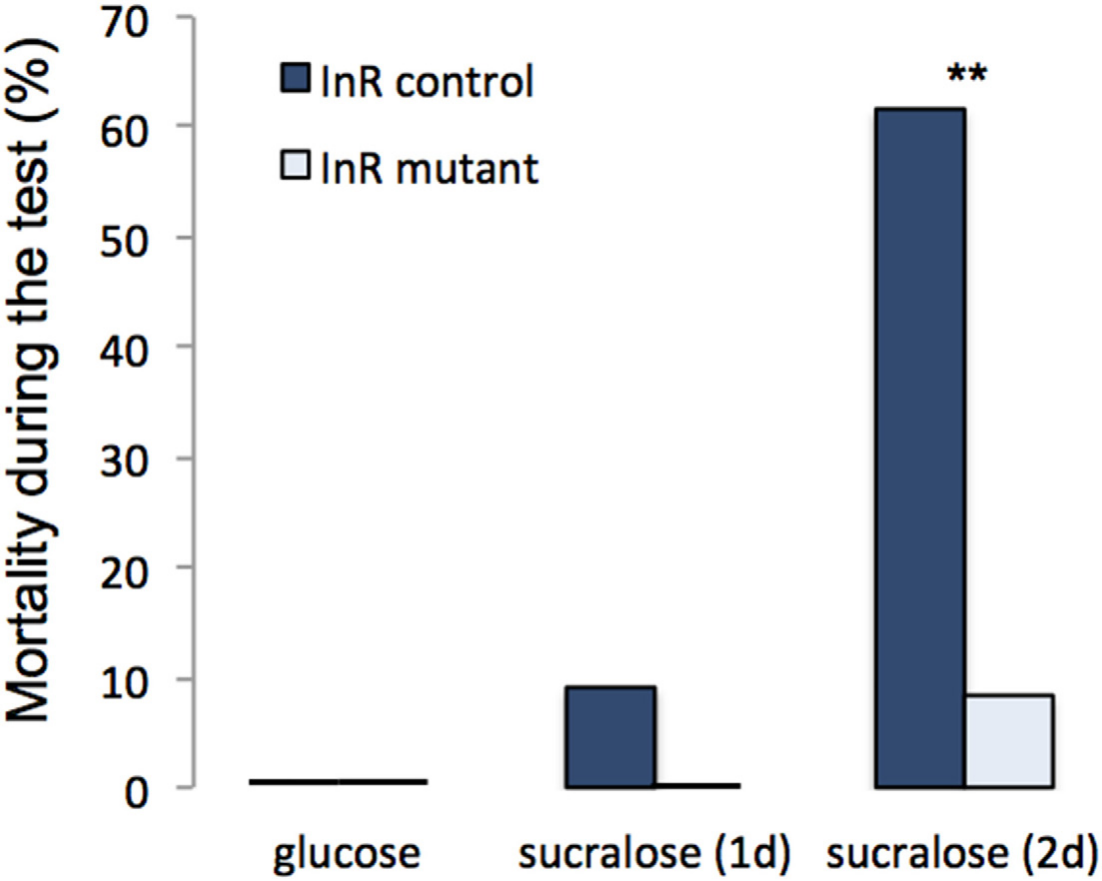
**Mortality of InR mutant and control flies during the feeding experiment when previously fed with glucose or with sucralose for 1 or 2 d**. Asterisks show a difference in the mortality between the two groups of flies (Chi^2^-test, χ = 7.37, *df* = 1, *p* = 0.007).

In contrast, a mutation of the InR affected feeding behavior depending on the fly previous diet (Figure 3B; interaction diet^*^genotype: *F* = 7.04, *df* = 1, *p* = 0.012). Indeed, InR mutants did not respond to nutrient deprivation (sucralose diet) by increased yeast consumption (Figure 3B). These flies also exhibited a higher yeast consumption rate when fed with glucose, at least for the first few hours of the test (Figure 3B). Moreover, insulin mutants were more resistant to starvation (Figure 4). When fed with sucralose for 2 days, 8.3% (1 out of 12) of the InR mutant flies died during the test period compared with 61.5% (16 out of 26) of control flies. When fed with glucose, all flies survived during this period (Figure 4; diet effect: Dev. = 17.91, *df* = 1, *p* < 0.001; genotype effect: Dev. = 10.72, *df* = 1, *p* = 0.001). When starved for 2 days at 24°C, WT flies had a higher mortality during the test than InR control flies (91.8%, *n* = 73, χ^2^ = 10.81, *df* = 1, *p* = 0.001) but the ones that survived showed a similar yeast consumption rate (0.53 ± 0.16 μL after 24 h for WT flies (*n* = 6); 0.46 ± 0.17 μL for InR control flies (*n* = 10); *W* = 29, *p* = 0.86).

## Discussion

The ecology of yeasts and *Drosophila* flies is strongly interconnected. Several *Drosophila* species forage on yeasts growing on the surface of decaying fruit during their larval and adult life. Deprivation of yeasts during larval development causes a drastic increase in mortality (Anagnostou et al., 2010; Becher et al., 2012). Volatile compounds produced by yeasts strongly attract *Drosophila* flies toward fermenting fruit that constitute a feeding, mating and egg-laying substrate (Becher et al., 2012). Reciprocally, when traveling from fruit to fruit, flies serve as a vector to spread yeasts (Spencer and Spencer, 1997; Becher et al., 2012).

Yeasts are a major source of proteins and lipids for *Drosophila* flies (Bos et al., 1976; Skorupa et al., 2008). Unbalanced diets, containing mainly sugars, lead to obese, short-lived flies (Skorupa et al., 2008). Adding yeast to unbalanced diets counteracts the negative effect of sugar (Skorupa et al., 2008). We therefore expected males fed only with glucose to feed on yeast. However, these flies did not increase yeast consumption in comparison to flies fed on rich cornmeal medium containing yeast. Dietary glucose consequently was sufficient to subsequently reduce yeast feeding. Yeast has an essential role in female fecundity as it stimulates egg production (Skorupa et al., 2008). Even though nutrition also affect male offspring production and competitive abilities (Fricke et al., 2008), yeast deprivation might not be as critical for males as it is for females. Indeed, even when starved for 3 days, *Drosophila* males are able to mate and produce as many offspring as fed males in non-competitive conditions (Lebreton et al., 2014). Consequently, a 3-day period of yeast deprivation might have only little effect on male fitness. This would explain why males only fed with glucose do not increase yeast consumption in our experiments.

Using sucralose, a non-metabolizable sweetener (Gordesky-Gold et al., 2008), we showed that the sugar effect was not due to a mechanical feedback of feeding or to the sweet taste of glucose, but rather due, either to its caloric value, or to the use of glucose as a signal molecule. *Drosophila* flies are able to detect the caloric content of sugars through a taste-independent process (Dus et al., 2011) and therefore learn to discriminate sugars with different nutritive values (Burke and Waddell, 2011; Stafford et al., 2012). A nutrient sensor in the fly brain directly detects an increase in hemolymph sugar level with a differential effect in starved and satiated flies (Miyamoto et al., 2012). It was hypothesized that hormonal pathways such as insulin signaling could cause this effect (Miyamoto et al., 2012).

The insulin pathway modulates food preference in *Drosophila* larvae (Wu et al., 2005a,b; Zhao and Campos, 2012). Our results show that the insulin pathway also influences yeast-feeding behavior in adults. An opposite effect of insulin was observed depending on the diet composition, suggesting that insulin could possibly regulate yeast consumption in response to both nutrient intake (glucose-fed) and deprivation (sucralose-fed flies). It has been previously reported that a null mutation of the fly InR substrate *chico* does not affect *Drosophila* food intake (Wong et al., 2009). It is possible that the insulin pathway mainly regulates food intake in response to changes in nutrient availability rather than modifying food intake as such.

Instead of increasing their yeast consumption as control flies do, sucralose-fed InR mutants keep a low feeding rate in a manner similar to control flies fed with glucose. Since insulin is released after a meal, it is unclear how the insulin pathway can be involved in increasing feeding upon starvation. However, in *Drosophila* adults, several ILPs are produced (Grönke et al., 2010; Nässel, 2012). They interact in a complex manner (Grönke et al., 2010) and activate a single receptor (InR). These different ILPs may have different roles and act differently to regulate feeding behavior. Indeed, when kept on low-calorie diet, flies lacking ILP2, 3, and 5 decrease food intake whilst flies lacking ILP7 increase food intake (Cognigni et al., 2011). It is important to note that ILP7 has been suggested to be more related to relaxin-like peptides rather than insulin-like peptides (Yang et al., 2008; Grönke et al., 2010; Veenstra, 2014) and might activate a different type of receptor (Veenstra, 2014). This suggests that these two insulin-like/relaxin-like pathways can have a context-dependent opposite effect on feeding behavior. The effect of insulin in the regulation of feeding behavior is not fully understood and our results highlight the complexity of this pathway. Further studies with different ILPs mutants and flies lacking insulin-producing cells are needed to get a better understanding of the mechanisms by which the insulin-signaling pathway regulates food intake. Mutations of the insulin pathway severely alter fly metabolism, resulting in an increase of circulating sugars and lipids (Teleman, 2010). It is therefore possible that the lack of response we observed in InR mutants after starvation is due to secondary effect of the altered metabolism rather than a direct effect of insulin of yeast consumption. Alternatively, an altered metabolism in InR mutants can lead to weaker flies when starved, resulting in a decrease in food-searching behavior and yeast consumption.

When fed with glucose, InR mutant flies did not suppress yeast consumption for the first few hours, suggesting that the insulin-signaling pathway may play a role in the regulation of feeding behavior also in response to nutrient intake. The failure to adjust food intake in response to food availability has previously been observed in *takeout* mutant flies (Meunier et al., 2007). The *takeout* gene encodes for a putative juvenile hormone (JH) binding protein and is involved in feeding behavior and metabolism. Similar to InR mutants in our experiments, *takeout* mutants do not increase their food intake after a period of starvation, but continue feeding when food is abundant (Meunier et al., 2007). The link between insulin and *takeout* is unclear, but the expression of both *ilp3* and *takeout* are regulated by the same calcium- and voltage-sensitive potassium channel (SLOB). SLOB mutant flies have decreased levels of *ilp3* and increased levels of *takeout* (Sheldon et al., 2011). Moreover, a recent study in the red flour beetle, *Tribolium castaneum*, suggested that JH regulates resistance to starvation by regulating the synthesis of ILP2 (Xu et al., 2013).

Interestingly, *takeout* mutants have a low resistance to starvation (Sarov-Blat et al., 2000). Unlike *takeout*, we report that InR mutants have an increased resistance to starvation, confirming previous findings (Broughton et al., 2005). Since InR mutants do not increase food consumption in response to starvation, they may not increase locomotor activity, which is associated with food foraging in WT flies (Lee and Park, 2004). Increasing locomotor activity induces a faster depletion of energy reserves and increases mortality. Increased starvation resistance has also been observed in flies with a disrupted AKH pathway (Lee and Park, 2004; Bharucha et al., 2008).

Both *takeout* and *Akhr* are expressed in gustatory neurons detecting sugars (Meunier et al., 2007; Bharucha et al., 2008). While *takeout* increases the sensitivity of these neurons after a period of starvation (Meunier et al., 2007), the effect of *Akhr* is unknown. We did not find any effect of AKHR on the regulation of yeast consumption in adult flies. Our results suggest that the role of AKH in response to starvation might be mainly restricted to the regulation of sugar and lipid metabolisms (mobilization and storage) rather than on the regulation of feeding behavior. This is in contrast to the findings of Bharucha et al. (2008) who reported that AKHR mutants have a reduced food intake after a period of starvation. A possible explanation of the discrepancy between the two studies is that Bharucha and colleagues focused on the first 30 min following starvation whereas we report here food intake 1–24h after starvation. It is therefore possible that AKHR mutants have a delayed initiation of feeding after starvation, the overall food consumption over longer periods being unaffected. The effect of AKH on the regulation of feeding behavior remains unclear and further studies will be needed to unravel the precise role of AKH on food intake.

In summary, the present study brings new insights into how the consumption of yeast, an ecologically relevant nutrient source, is modulated. Although dietary glucose is incomplete with respect to contents of essential nutrients, it seems to be sufficient to decrease yeast consumption in adult males. Insulin was found being involved in the regulation of yeast feeding whereas no effect of AKH was detected. These results address the question of the precise role of insulin and AKH pathway in the regulation of *Drosophila*-yeast interactions.

### Conflict of interest statement

The authors declare that the research was conducted in the absence of any commercial or financial relationships that could be construed as a potential conflict of interest.
